# The Impact of the Geometrical Structure of the DNA on Parameters of the Track-Event Theory for Radiation Induced Cell Kill

**DOI:** 10.1371/journal.pone.0164929

**Published:** 2016-10-19

**Authors:** Uwe Schneider, Fabiano Vasi, Jürgen Besserer

**Affiliations:** 1 Department of Physics, Science Faculty, University of Zürich, Zürich, Switzerland; 2 Radiotherapy Hirslanden, Witellikerstrasse 40, CH-8032, Zürich, Switzerland; ENEA Centro Ricerche Casaccia, ITALY

## Abstract

**Background and Purpose:**

When fractionation schemes for hypofractionation and stereotactic body radiotherapy are considered, a reliable cell survival model at high dose is needed for calculating doses of similar biological effectiveness. An alternative to the LQ-model is the track-event theory which is based on the probabilities for one- and two two-track events. A one-track-event (OTE) is always represented by at least two simultaneous double strand breaks. A two-track-event (TTE) results in one double strand break. Therefore at least two two-track-events on the same or different chromosomes are necessary to produce an event which leads to cell sterilization. It is obvious that the probabilities of OTEs and TTEs must somehow depend on the geometrical structure of the chromatin. In terms of the track-event theory the ratio *ε* of the probabilities of OTEs and TTEs includes the geometrical dependence and is obtained in this work by simple Monte Carlo simulations.

**Materials and Methods:**

For this work it was assumed that the anchors of loop forming chromatin are most sensitive to radiation induced cell deaths. Therefore two adjacent tetranucleosomes representing the loop anchors were digitized. The probability ratio *ε* of OTEs and TTEs was factorized into a radiation quality dependent part and a geometrical part: *ε*_*=*_ *ε*_*ion ∙*_ *ε*_*geo*_. *ε*_*geo*_ was obtained for two situations, by applying Monte Carlo simulation for DNA on the tetranucleosomes itself and for linker DNA. Low energy electrons were represented by randomly distributed ionizations and high energy electrons by ionizations which were simulated on rays. *ε*_*ion*_ was determined for electrons by using results from nanodosimetric measurements. The calculated *ε* was compared to the *ε* obtained from fits of the track event model to 42 sets of experimental human cell survival data.

**Results:**

When the two tetranucleosomes are in direct contact and the hits are randomly distributed *ε*_*geo*_ and *ε* are 0.12 and 0.85, respectively. When the hits are simulated on rays *ε*_*geo*_ and *ε* are 0.10 and 0.71. For the linker-DNA *ε*_*geo*_ and *ε* for randomly distributed hits are 0.010 and 0.073, and for hits on rays 0.0058 and 0.041, respectively. The calculated *ε* fits the experimentally obtained *ε = 0*.*64±0*.*32* best for hits on the tetranucleosome when they are close to each other both, for high and low energy electrons.

**Conclusions:**

The parameter *ε*_*geo*_ of the track event model was obtained by pure geometrical considerations of the chromatin structure and is 0.095 ± 0.022. It can be used as a fixed parameter in the track-event theory.

## Introduction

The linear-quadratic (LQ) model is currently the model of choice for applications in radiation oncology [1]. However, the LQ dose-response curve for cell survival bends continuously on the log-linear plot which cannot describe the experimental data obtained in many studies. The dose-response curves of these experimental data exhibit a more exponential decrease in survival, i.e. a more straight line on the log-linear plot [2–4].

Several alternative methodologies which describe cell kill exhibiting an exponential decrease at large dose were developed and are briefly summarized in the following. One of the first approaches was the “single-hit multi-target formula” [5]. For this model it is assumed that a cell is deactivated only when at least *n* targets in the cell are hit. The major drawback of this model is that the cell-survival curve exhibits a vanishing gradient at low dose which is not in accordance with experimental data. Another model is the “universal survival curve” [6] which is the combination of a LQ model with a linear extension at large dose. This model is essentially an empirical description which fits clinical data well, but is not based on a mechanistic understanding of the underlying processes which lead to cell kill. Recently Ekstrand [7] re-examined the Hug-Kellerer [8] model of cell survival and established the relationship between this model and the LQ-model. This model fits well published cell survival curves over a wide dose range. However, this is achieved by introducing a third fitting parameter. Lately, a simple track-event model of cell survival from Poisson statistics was developed [9]. The model is evolved from merely a few basic assumptions and it is based on only two parameters. In this model [9] the cell survival curve is exponential at high dose and has a finite gradient of cell survival in the limit of zero dose. The model of Besserer and Schneider [9] assumes that the sensitive targets are chromosomal DNA. Furthermore, it is assumed that double strand breaks (DSBs) are the significant results of energy depositions. All single-strand breaks are assumed to be non-lethal. Moreover, it was assumed that one DSB is non-lethal and an event was defined”by two lethal DSBs on the same or different chromosomes” [9]. One or more events are always lethal due to direct lethal damage or chromosomal aberrations. The track-event model distinguishes two types of events: one-track events (OTE) and two-track events (TTE). Two or more DSBs, on the same or on different chromosomes, caused by only one track constitute an OTE. Two DSBs caused by different tracks constitute a TTE. It is called TTE since at least two tracks are needed for a lethal event.

If it is assumed that OTEs and TTEs are statistically independent events, the probabilities for those events can be obtained from Poisson statistics. The main difference of the approach used here to already published work is, that the probabilities are obtained for surviving cells and not for sterilized cells [9]. The cell survival probability is then simply given by:

$$S=\left(1+qD\right)\cdot e^{-\left(q+p\right)D},$$ (1)

where *p* and *q* are the probabilities for OTEs and TTEs, respectively.

An important difference of this model compared to the molecular theory of Chadwick and Leenhouts [10] is that all lethal events are based on direct DSBs.

An extension of the model was developed to include a repair-probability for two DSBs [11]. The equation for the total probability for cell survival with second-order repair is then given by:

$$S=e^{-\left(1+\varepsilon \right)\cdot q\cdot D}\left(1+D\cdot q\cdot \left(1+\varepsilon \cdot R\right)+D^{2}\cdot q^{2}\cdot \left(\frac{R}{2}+\varepsilon \cdot R\right)+D^{3}\cdot q^{3}\cdot \frac{R^{2}\cdot \varepsilon }{2}\right),$$ (2)

where *R* represents the probability for either repairing two DSBs or the formation of non-lethal chromosomal aberrations and *ε = p/q*. Further it was assumed that the absolute probabilities *p* and *q* will depend on the distribution of the ionizing events and the three-dimensional structure of the cell nuclei. Therefore *ε* depends on the radiation quality and cell geometry, such as the interphase chromosome territories, the number of chromosomes, the dimension of the chromosome territories, the looping probabilities of the chromatin fiber and many others. As a consequence the ratio of *p* and *q* should, besides radiation quality (*ε*_*ion*_), dependent solely on the fundamental organization of the chromatin fiber (*ε*_*geo*_):

$$\varepsilon \;=\epsilon_{ion}\;\cdot \;\varepsilon_{geo}$$ (3)

Therefore it can be hypothesized that all cells that have the same basic chromatin structure in common, are subject to the same *ε* when they are irradiated with the same radiation quality. Since the tetranucleosome is the basic component of the chromatin fiber, *ε* for one particle/radiation type should solely depend on the distribution of the DNA in the tetranucleosome. Therefore, *ε*_*geo*_ can be determined in principle from the geometrical structure of the tetranucleosome.

The aim of this work is the determination of *ε*_*geo*_ by simulating particle tracks through the basic geometry of the DNA and counting the resulting OTEs and TTEs. The DNA-geometry has been digitized by using the structure of a tetranucleosome according to Schalch et al. [12]. In this work *ε*_*geo*_ is simulated for a loop contact defined by two tetranucleosomes touching or being in close vicinity to each other. In this scenario a chromosomal aberration is the result of two DSBs on two segments of the loop, i.e. at least one DSB on each of the two tetranucleosomes. Further, the produced DSBs are analyzed separately by obtaining *ε*_*geo*_ for OTEs and TTEs on the whole tetranucleosome and on the linker-DNA only. Finally *ε*_*ion*_ is determined by using results of measured cluster size distributions for electrons from nanodosimetry. The resulting calculated *ε* is then compared to *ε* obtained from fits to human cell survival data for photon radiation.

## Materials and Methods

### Structure of a Tetranucleosome

**Fig 1:**
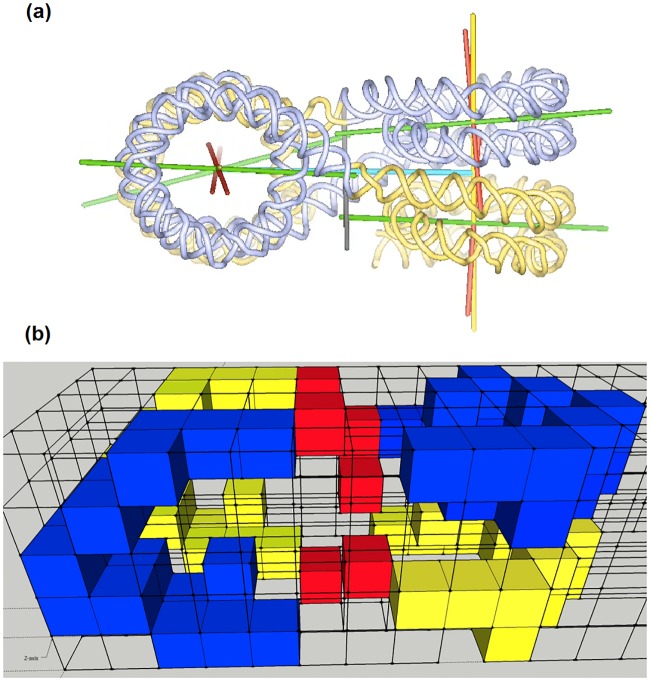
Observed and digitized structure of the tetranucleosome. Different views of the structure of the tetranucleosome in (a) as obtained by the work of Schalch et al. [9] and in (b) as it was digitized for the presented Monte Carlo work. Please note that for digitization the axis of the nucleosomes were not inclined, but assumed to be parallel. Blue and yellow colors indicate voxels which belong to the tetranucleosome. The linker DNA is illustrated by red voxels.

**Fig 2:**
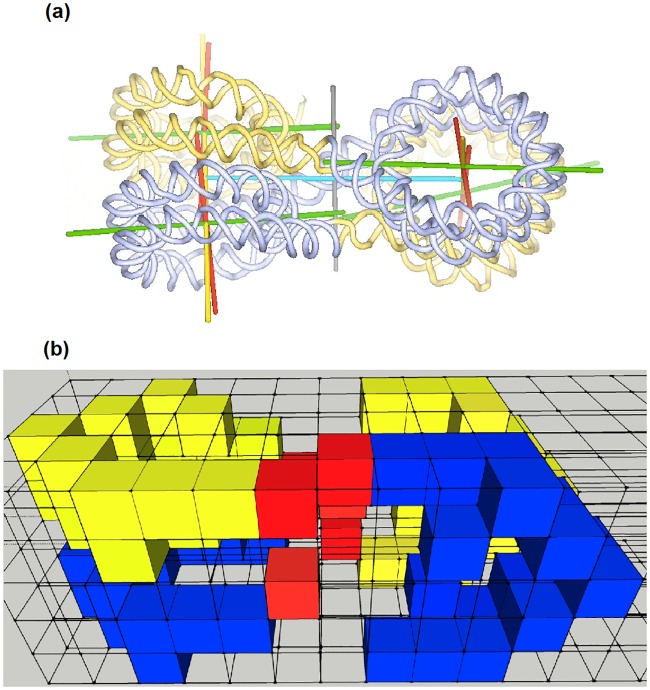
Observed and digitized structure of the tetranucleosome. Another view of the structure of the tetranucleosome in (a) as obtained by the work of Schalch et al. [9] and in (b) as it was digitized for the presented Monte Carlo work. Please note that for digitization the axis of the nucleosomes were not inclined, but assumed to be parallel. Blue and yellow colors indicate voxels which belong to the tetranucleosome. The linker DNA is illustrated by red voxels.

Eight histone molecules form an octomeric histone core around which the DNA is wrapped to form a 11 nm fiber [13, 14]. Nucleosomes appear rarely in this form since the chromatin is mostly in its more compacted form in which nucleosomal arrays are folded into chromatin fibers [15, 16]. The compact chromatin fibers have a diameter of approximately 30 nm. The structure of the nucleosome core was obtained at high resolution by Davey et al. [17]. The organization of the nucleosomes inside the chromatin fiber was determined by Schalch et al. [16]. The nucleosome is between 157 and 240 base pairs (bp) long. The DNA is wrapped around four histone proteins (H2A, H2B, H3 and H4) and the linker histone protein (H1). The tetranucleosome determined by Schalch et al. [16] is composed of two stacks of nucleosome cores (N1 and N2). The two stacks are connected by three linkers (LB, LB’, and LS) of DNA (see Figs 1a and 2a). From center to center the distance between the two stacks is 14.61 nm. The two stacks are rotated by -71.3° with respect to each other. The two nucleosome cores in the same stack are separated by 5.76 nm. The dimension of the tetranucleosome is 12 x 15 x 25 nm^3^. The tetranucleosome consists of four repeat lengths, each with a length of 147 bp. The linkers have a length of 20 bp [16].

**Fig 3:**
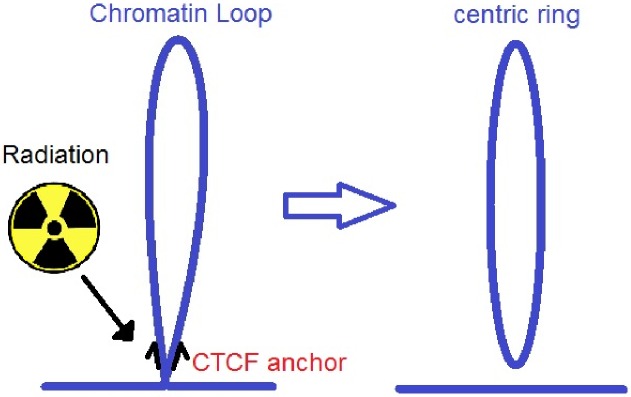
Chromatin loops. Many loops in chromatin demarcate contact domains. Usually the loops are anchored at a pair of convergent CTCF binding sites. It is hypothised that radiation is most effective by producing DSBs at the anchor locations by producing rings.

Chromatin forms loops. These are defined as stretches of genomic sequence on the same chromosome which are in closer physical proximity to each other than to intervening sequences [18]. Chromatin loops correlate with gene activation and show conservation across cell types. Loop anchors usually bind the insulator protein CTCF (see Fig 3) [19].

The tetranucleosome is the largest repeating structure of the DNA organization and it was therefore decided to digitize it by using the software package PVWave (PV-Wave Advantage, PV-Wave Command Language, Version 9.01—Numerics, Inc—2008). The distance per bp is 0.34 nm and since one nucleosome consists of 147 bp it has a length of 147 x 0.34 nm = 49.98 nm. The voxel size in the model was chosen to be 2 nm, which represents the width of the double helix. The fiber is 11 nm in diameter which leads to a packing ratio of 49.98/11 = 4.54. In the implementation the size of the 11 nm fiber is simulated by 5 voxels and therefore a packing ratio of 49.98/10 achieved. The 147 bp are assumed to build one complete ring. Hence, the base pair density can be calculated by:

$$\frac{147\;bp}{\pi \cdot 10\;nm}=4.68\frac{bp}{nm}$$ (4)

**Fig 4:**
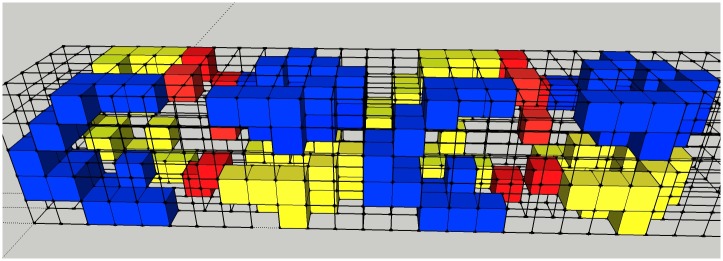
Simulation geometry. View of the simulation geometry of the anchor location of a chromatin loop by placing two tetranucleosomes adjacent to each other. Blue and yellow colors indicate voxels which belong to the tetranucleosome. The linker DNA is illustrated by red voxels.

As the voxels have a side length of 2 nm and 2 nm x 4.68 bp/nm = 9.36 bp, one voxel possesses approximately 9 base pairs. Therefore in the simulation a tetranucleosome consists of 144 base pairs. Figs 1a and 2a show the representation of the tetranucleosome of Schalch et al [16] and the corresponding digitized model is shown in Figs 1b and 2b. It is assumed that the DNA is most vulnerable where the chromatin loops make contact (Fig 3). Therefore, to simulate this, two tetranucleosomes are placed next to each other in such a way that one tetranucleosome represents the start of the loop and the other the end of the loop (shown in Fig 4).

### Simulation of *ε*_*geo*_

It is assumed that the sensitive target of radiation is the chromosomal DNA. For two reasons it is supposed that the most sensitive structures of a cellular DNA are the anchors of chromatin loops. Firstly, the tetranucleosomes of the anchors are in close physical proximity as they are usually linked to each other through CTCF proteins [19]. This proximity guarantees that the two tetranucleosomes have the possibility to interact with each other. As a consequence, when DNA strands are broken at this position, they have a higher chance to misrepair by joining DNA strands from tetranucleosomes on different loop branches. Therefore if DSBs occur at the anchor of a loop the probability to build a centric ring is higher than elsewhere (Fig 3). As mentioned above these centric rings as well as the dicentric aberrations probably are a major reason for cell sterilizations. We hypothesized that the linkers are more vulnerable to the formation of DSBs and therefore of chromosomal aberrations as they are not packed as much as the DNA of the tetranucleosomes. In addition, they are not wrapped around histones which can have a destabilizing effect. Therefore, in a second simulation the linkers were considered individually. An important difference of the presented loop-model compared to the work of Friedrich et al [20] is that in this work all relevant DSBs happen in the vicinity of the loop anchors. In the approach of Friedrich et al [20] all DSBs on the loop are examined by distinguishing between isolated and clustered DSBs.

The Monte Carlo simulations were performed using the software package PVWave (PV-Wave Advantage, PV-Wave Command Language, Version 9.01—Numerics, Inc—2008). The assumptions regarding energy deposition are adopted from Besserer and Schneider [9, 11]. Ionizing radiation is assumed to be isotropic. The structure to be irradiated which is shown in Fig 4 is placed in the center of a sphere of 32 nm diameter. Randomly distributed entrance and exit coordinates of infinitesimal small rays were determined on the surface of the sphere. A straight line connection of entrance and exit coordinates represented the trajectory of the ray. Every voxel which was touched by the ray was assumed to be a hit. A DSB was occurring if a voxel which contained DNA was hit. Events were created by the two processes: (i) A one-track event is counted when the simulated ray intersects at least one voxel containing DNA of tetranucleosome A and one in tetranucleosome B. (ii) A two-track event is counted when two independent rays hit a DNA-voxel in tetranucleosome A and tetranucleosome B. The number of TTEs in tetranucleosome A and B were calculated by subtracting from the total number of hits in A and B the number of one-track events, respectively. Single strand breaks were assumed to be non-lethal and were not simulated. The number of simulations was chosen such that the statistical error was negligible (see supporting information “S1 Table”).

We compare in this work the simulated OTE to TTE ratio with the results from cell survival curves obtained with photon radiation. The electrons which are responsible for energy deposition follow usually not straight lines. We therefore estimated the maximum lateral deflection of electrons of different energies when they are constrained by entrance and exit coordinates through the 32 nm sphere by using published elastic scattering cross sections [21–24]. It was found that the electrons scatter laterally not more than 2 nm (voxel size) as long as the electron energy is above 1000 eV. Thus the tracks of electrons of energies larger than 1000 eV are confined to a narrow cylindrical region with 2 nm distance from the straight line connection of entrance and exit coordinate. For very low energy electrons it was assumed that the ionizations occur randomly in the simulation geometry. Thus, instead of using a ray tracing method the ionization-events were randomly distributed and OTEs and TTEs were counted and analyzed as described above (see supporting information “S2 Table”). For electron energies between 30 eV and 1000 eV the distribution of the ionizations is assumed to fall in between the two extreme cases of randomly distributed hits and hits that lie on straight rays.

### Determination of *ε*_*ion*_ and comparison with cell survival data

In a previous work [11] *ε* was fitted to 42 sets of cell survival data of human cell lines that were irradiated with Co-60, Cs-137, or X-ray radiation of at least 220 keV energy. The resulting averaged *ε* was found to be 0.64 with an error of 0.32 (one standard deviation) [11]. In order to compare the geometrical obtained *ε*_*geo*_ of this work to the fitted *ε* it is necessary to estimate *ε*_*ion*_.*ε*_*ion*_ depends by definition on the distribution of the ionisations of the electrons produced by the photon beam. As described above, DSBs can be produced by OTEs and TTEs. As OTEs are created by one particle track they can be represented by the nanodosimetric quantity *F*_*2*_, which is the probability of two or more ionisations in a volume comparable to our voxel size. On the other hand TTEs are created by two independent ionisations and are hence represented by *p*_*1*_^*2*^, where *p*_*1*_ is the probability for a single ionization. *ε*_*ion*_ is then defined by:

$$\epsilon_{ion}=\;{\left(\frac{F_{2}}{p_{1}{}^{2}}\right)}^{2}.$$ (5)

**Fig 5:**
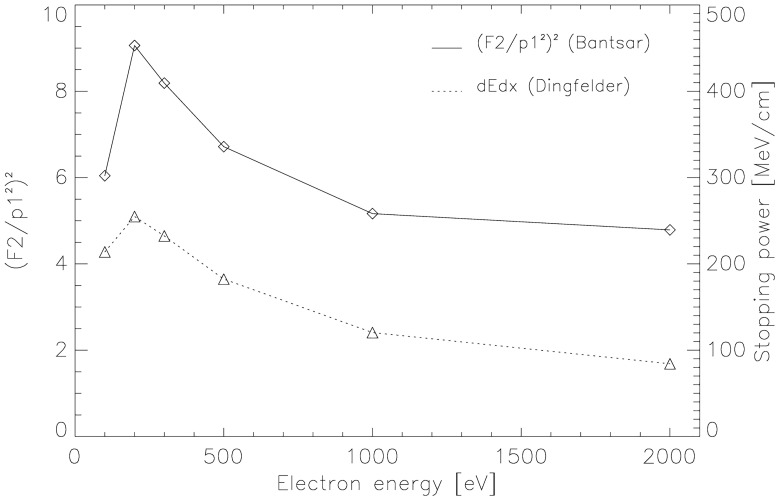
*ε*_*ion*_ and electron stopping power. Plot of *ε*_*ion*_ as a function of electron energy as the solid line (left axis). The dotted line (right axis) illustrates the electron stopping power as a function of energy.

The probabilities in (Eq 5) are squared, as in our geometry two independent DSBs are needed, one on tetranucleosome A and one on tetranucleosome B. The probabilities *F*_*2*_ and *p*_*1*_ were taken from nanodosimetric experiments with electrons of various energies from Bantsar [25–27]. In Fig 5 *ε*_*ion*_ is plotted as a function of electron energy as the solid line. The average *ε*_*ion*_ over all electron energies is 6.7. However, not all electrons deposit the same energy into DNA which is reflected by the variation of electron stopping power with energy illustrated as the dotted line in Fig 5. Electron stopping powers were taken from Dingfelder et al [28]. The functional dependence of the electron stopping power as a function of energy was used to calculate a stopping power weighted average *ε*_*ion*_ = 7.1 which was used for further analyses.

## Results

**Fig 6:**
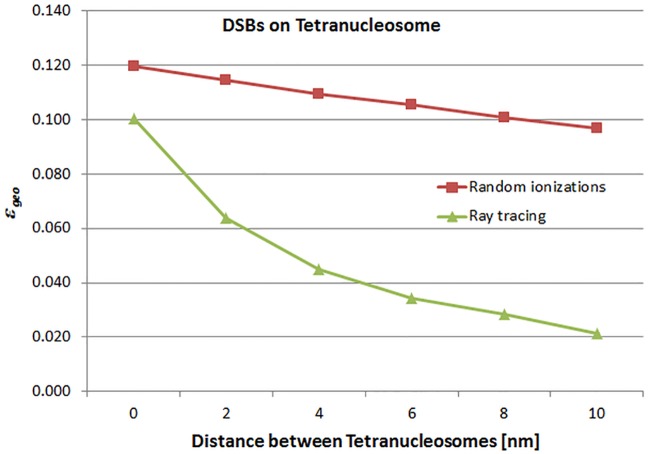
*ε*_*geo*_ for tetranucleosomal DNA. Illustration of the behavior of the *ε*_*geo*_ for increasing distances between tetranucleosomes A and B (data listed in Table 1) for the simulation using hits on the whole tetranucleosomal DNA. The squares mark the results from randomly distributed hits and the triangles from hits on straight rays.

**Fig 7:**
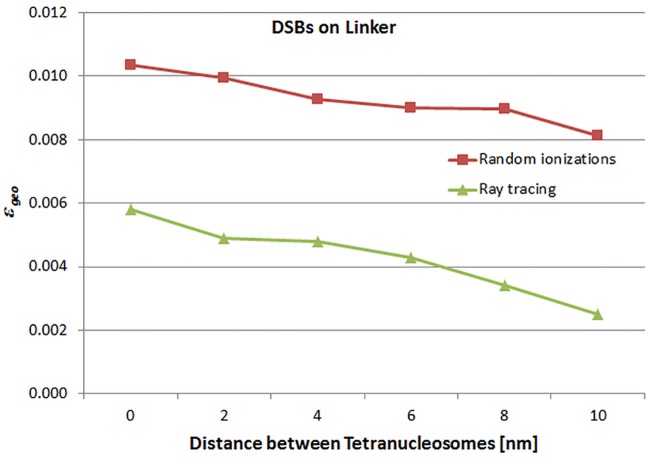
*ε*_*geo*_ for linker DNA. Illustration of the behavior of *ε*_*geo*_ for increasing distances between tetranucleosomes A and B (data listed in Table 1) for the simulation using hits on the linker DNA. The squares mark the results from randomly distributed hits and the triangles from hits on straight rays.

Figs 6 and 7 show the behavior of *ε*_*geo*_ for increasing distances between tetranucleosomes A and B (data listed in Table 1). Fig 6 shows the results of the simulation using hits on the whole tetranucleosomal DNA as opposed to Fig 7, where only the hits on linker DNA were counted. Furthermore, simulations were carried out for randomly distributed ionizations representing low energy electrons and ionizations on straight rays representing high energy electrons, which are shown as the squares and triangles, respectively. *ε*_*geo*_ is decreasing with increasing distance between the tetranucleosomes, i.e. with increasing distance between the two tetranucleosomes the number of OTEs is reduced and the number of TTEs is increased. When tetranucleosome A is in direct contact with tetranucleosome B and the hits are distributed randomly *ε*_*geo*_ is 0.119 and 0.100 if the hits are distributed along rays. For the linker-DNA *ε*_*geo*_ is for randomly distributed hits and for hits on rays 0.0103 and 0.0058, respectively. Detailed data can be found in the supplemental “S1 and S2 Tables”.

**Table 1:** Simulated *ε*_*geo*_ as a function of the distance between the two Tetranucleosomes.

Distance between Tetranuleosomes in nm	*ε*_*geo*_			
	Random ionizations		Ray tracing	
	on Nucleosome	on Linker	on Nucleosome	on Linker
0	0.119	0.0103	0.100	0.0058
2	0.114	0.0099	0.064	0.0049
4	0.109	0.0093	0.045	0.0048
6	0.105	0.0090	0.034	0.0043
8	0.101	0.0089	0.028	0.0034
10	0.097	0.0081	0.021	0.0025

The values are given for randomly and straight line distributed ionizations and for hits on the whole Tetranucleosome or linker DNA, respectively.

In Table 2 the total *ε* = *ε*_*ion*_ *∙ ε*_*geo*_ is listed for the same configurations than in Table 1 and with *ε*_*ion*_ = 7.1. The determined *ε* can be compared to the *ε* resulting from the cell survival data. Bold numbers in Table 2 indicate those *ε* which are in accordance with the results from fits to human cell survival data within fitting errors. A mean ${\bar{\varepsilon }}_{geo}$ can be calculated by averaging *ε*_*geo*_ in Table 1 at the corresponding bold positions of Table 1. The resulting ${\bar{\varepsilon }}_{geo}=\;0.095\;\pm \;0.022$.

**Table 2:** Total *ε* = *ε*_*ion*_ *∙ ε*_*geo*_ as a function of the distance between the two Tetranucleosomes.

Distance between Tetranuleosomes in nm	*ε* = *ε*_*ion*_ *∙ ε*_*geo*_			
	Random ionizations		Ray tracing	
	on Nucleosome	on Linker	on Nucleosome	on Linker
0	**0.845**	0.073	**0.710**	0.041
2	**0.809**	0.070	**0.454**	0.035
4	**0.774**	0.066	**0.320**	0.034
6	**0.746**	0.064	0.241	0.031
8	**0.717**	0.063	0.199	0.024
10	**0.689**	0.058	0.149	0.018

The values are given for randomly and straight line distributed ionizations and for hits on the whole Tetranucleosome or linker DNA, respectively. Bold numbers indicate those *ε* which are in accordance with the results from fits to cell survival data within fitting errors.

## Discussion

In this work it was shown that the geometry of the chromatin structure can have a significant impact on the cell survival curve. It should be noted, however, that the results of this work were obtained at a specific location in the chromatin: at the position of the anchors where chromatin forms loops. This was done, as we believe that a chromatin loop is in particular sensitive to DSBs and thus ionizing radiation is most effective at the anchor positions (high probability for forming centric rings). We know that this is an assumption which has currently no experimental evidence and should be therefore viewed as an example of how chromatin structure can impact radiation induced cell death.

The parameter *ε* of the track event model which is the ratio of the probabilities of one- and two-track events is assumed to depend on radiation quality and chromatin structure. As *ε* is a probability-ratio it is not necessary to obtain the absolute probabilities for OTEs and TTEs separately. As only the ratio of the two quantities is required, simple Monte Carlo methods can be used to determine the dependence of *ε* on the chromatin structure.

The results of this work which are listed in Table 2 and were compared to the recently obtained *ε = 0*.*64 ± 0*.*32* from fits to human cell survival data [11]. The *ε* obtained from hits on the whole tetranucleosomal DNA are an order of magnitude larger than the *ε* which result from hits on linker DNA only. For both, randomly distributed hits (low energy) and ray tracing (high energy), the *ε* determined for the tetranucleosome are in accordance with the fits to the cell survival data. For the ray tracing simulation which represents electron energies larger than 1000 eV the agreement is in particular good when the two nucleosomes are close to each other. This finding supports the assumption that loop anchors, where the tetranucleosomes are touching, are radiation sensitive structures.

Although linker DNA is, in contrast to tetranucleosomal DNA, not fixed to histones and from a naive perspective more sensitive to misrepair of DSBs, the comparison of the results from this work to the fits of the cell survival data do not support this view.

A limitation of this work is that only one specific geometrical situation was analysed: DSBs on adjacent tetranucleosomes at loop anchors of chromatin. It could be that the investigated detail of the nucleosome structure might not be the most radiation sensitive part of the chromatin structure. It could be also wrong that DSBs must occur on different strands of tetranuceosomes. Perhaps complex DSBs in only one tetranucleosome can lead also to cell sterilization.

## Conclusions

The parameter *ε*_*geo*_ of the track event model was obtained by pure geometrical considerations of the chromatin structure and is 0.095 ± 0.022. It can be used as a fixed parameter in the track-event theory.

Future work should include the whole chromatin structure in Monte Carlo simulations and not only the anchor point of chromatin forming loops. For the determination of the impact of different radiation qualities on *ε* Monte Carlo simulations exhibiting the complete track structure can be performed.

## Supporting Information

S1 Table Output file of the Monte Carlo simulation (ray tracing). List of the simulated raw data for different distances between the two tetranucleosomes. The calculated number of hits are listed for OTEs and TTEs and for the location of the hit (nucleosome-DNA or linker-DNA).
(TXT)

S2 Table Output file of the Monte Carlo simulation (randomly distributed hits). List of the simulated raw data for different distances between the two tetranucleosomes. The calculated number of hits are listed for OTEs and TTEs and for the location of the hit (nucleosome-DNA or linker-DNA).
(TXT)
